# Disease mutations reveal residues critical to the interaction of P4-ATPases with lipid substrates

**DOI:** 10.1038/s41598-017-10741-z

**Published:** 2017-09-05

**Authors:** Rasmus H. Gantzel, Louise S. Mogensen, Stine A. Mikkelsen, Bente Vilsen, Robert S. Molday, Anna L. Vestergaard, Jens P. Andersen

**Affiliations:** 1 Department of Biomedicine, Aarhus University, Ole Worms Allé 4, Bldg. 1160, DK-8000 Aarhus C, Denmark; 2 Department of Biochemistry and Molecular Biology, University of British Columbia, Vancouver, British Columbia V6T 1Z3 Canada; 3 Department of Ophthalmology and Visual Sciences, Centre for Macular Research, University of British Columbia, Vancouver, British Columbia V6T 1Z3 Canada; 4 Laboratory for Immuno-Endocrinology, Department of Biomedical Sciences, University of Copenhagen, DK-2200 Copenhagen N, Denmark

## Abstract

Phospholipid flippases (P_4_-ATPases) translocate specific phospholipids from the exoplasmic to the cytoplasmic leaflet of membranes. While there is good evidence that the overall molecular structure of flippases is similar to that of P-type ATPase ion-pumps, the transport pathway for the “giant” lipid substrate has not been determined. ATP8A2 is a flippase with selectivity toward phosphatidylserine (PS), possessing a net negatively charged head group, whereas ATP8B1 exhibits selectivity toward the electrically neutral phosphatidylcholine (PC). Setting out to elucidate the functional consequences of flippase disease mutations, we have identified residues of ATP8A2 that are critical to the interaction with the lipid substrate during the translocation process. Among the residues pinpointed are I91 and L308, which are positioned near proposed translocation routes through the protein. In addition we pinpoint two juxtaposed oppositely charged residues, E897 and R898, in the exoplasmic loop between transmembrane helices 5 and 6. The glutamate is conserved between PS and PC flippases, whereas the arginine is replaced by a negatively charged aspartate in ATP8B1. Our mutational analysis suggests that the glutamate repels the PS head group, whereas the arginine minimizes this repulsion in ATP8A2, thereby contributing to control the entry of the phospholipid substrate into the translocation pathway.

## Introduction

P_4_-ATPases, known as flippases, are lipid pumps that contribute to the phospholipid asymmetry of cellular membranes, thereby being crucial to a number of physiologically important functions. Fueled by ATP, the flippases translocate specific phospholipids such as phosphatidylserine (PS), phosphatidylethanolamine (PE), or phosphatidylcholine (PC) from the exoplasmic to the cytoplasmic membrane leaflet^1–9^. Flippases constitute the largest subfamily of the P-type ATPases, with 14 different human genes of 36 human P-type ATPase genes in total. The phospholipid specificity seems to differ among the human flippases, but is well characterized only for some of the family members, such as the PS flippases ATP8A1 (PS > PE, no PC), ATP8A2 (PS > PE, no PC), ATP11A (PS > PE, no PC), and ATP11C (PS > PE, no PC), while being somewhat controversial for ATP8B1 (PC, PS?) and unknown for several others^1–8^. ATP8B1 was originally considered a PS flippase^1^, but recent studies in HeLa and CHO-K1 cells indicate that ATP8B1 (as well as ATP8B2 and ATP10A) selectively translocate phosphatidylcholine (PC)^8, 9^. The difference in charge of the head group (PS negatively charged, PE and PC neutral), but probably also its size and other chemical characteristics, may form the basis for the selectivity.

P_4_-ATPase gene mutations result in a broad variety of pathophysiological conditions. Severe neurological manifestations have been reported for mutations of the neuronal flippase ATP8A2^10, 11^. The largest group of patients with flippase defects suffer from the intrahepatic cholestasis diseases PFIC1 (progressive familial intrahepatic cholestasis type 1) and BRIC1 (benign recurrent intrahepatic cholestasis type 1) caused by mutation of the liver-expressed ATP8B1^1, 12, 13^. BRIC1 is the mildest variant, with an onset in childhood of recurring episodes of cholestasis, but without detectable liver damage under remission. The more debilitating PFIC1 usually debuts within the first year of life and progresses to end-stage liver disease during the adolescence. The underlying molecular pathophysiology of these disorders is not clear, but the accumulation of PC or PS (depending on what the lipid substrate of ATP8B1 actually is) in the outer leaflet caused by reduced ATP8B1 activity might lead to shedding of membrane protrusions into the bile and inactivation of the bile transporter ABCB11^1, 8^.

**Figure 1:**
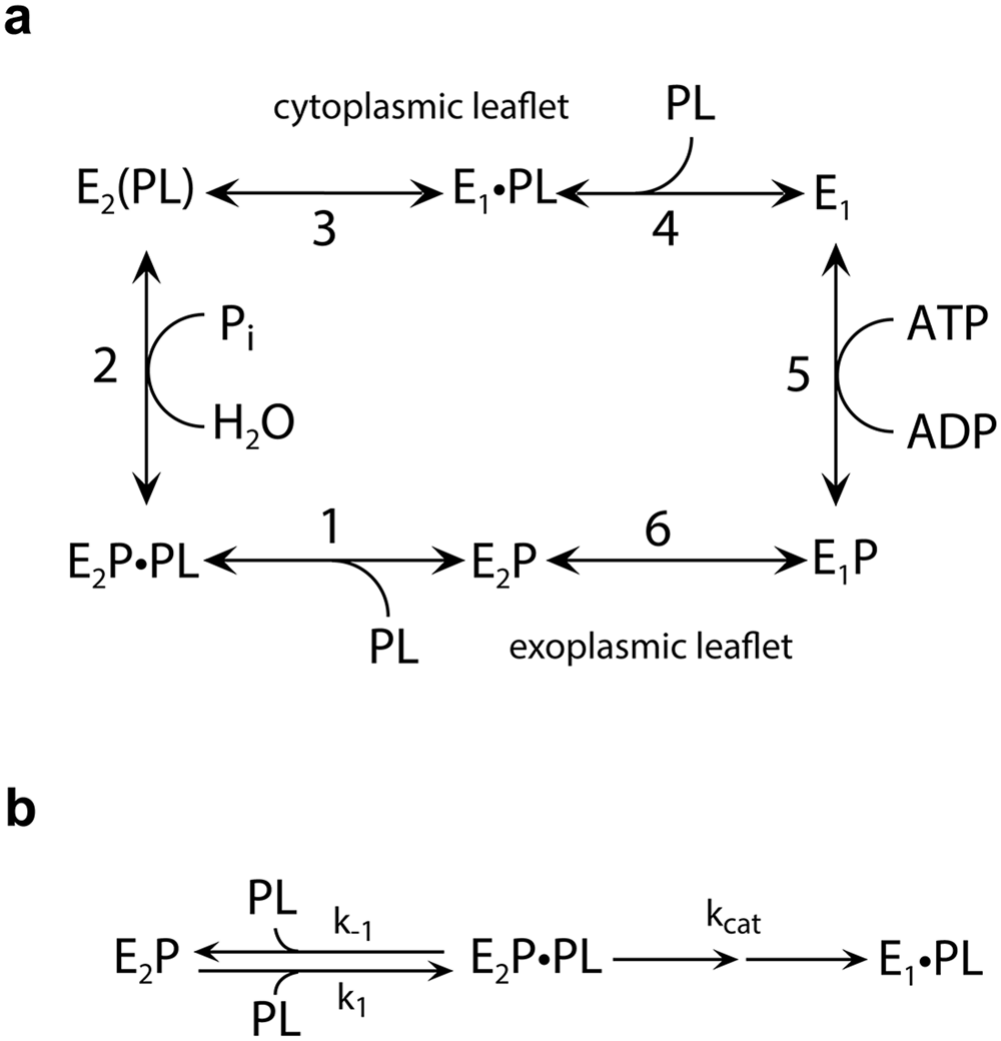
Flippase reaction cycle. (**a**) The scheme is based on functional similarities to the ion transporting P-type ATPases^15^. E_1_, E_1_P, E_2_P, and E_2_ are the major enzyme conformational states, the “P” indicating covalently bound phosphate. Reactions are numbered 1–6. The phospholipid substrate, PL, enters the cycle from the exoplasmic leaflet of the lipid bilayer by binding to the E_2_P phosphoenzyme (Reaction 1), thereby stimulating the dephosphorylation (Reaction 2) and release of the lipid toward the cytoplasmic leaflet (Reaction 4) as a consequence of the E_2_ to E_1_ transition (Reaction 3). The transition to E_1_ allows the enzyme to become rephosphorylated (Reaction 5). The stimulation of dephosphorylation by the lipid substrate is analogous to the activation of Na^+^,K^+^-ATPase dephosphorylation by K^+^ binding to E_2_P from the extracellular side of the membrane^2^. In further analogy with the modes of binding and transport of K^+^ by the Na^+^,K^+^-ATPase, the parentheses in E_2_(PL) indicate that the PL is bound to the protein in an occluded state, where the protein conformational change to E_1_ is required before the PL can be released toward the cytoplasmic side. In E_2_P·PL and E_1_·PL the · indicates that PL in these complexes is able to dissociate (to the exoplasmic leaflet and the cytoplasmic leaflet, respectively). (**b**) Rate constants relating to the rate-limiting dephosphorylation reaction and subsequent conformational change. Under the experimental conditions applied here, the dephosphorylation is essentially irreversible, because the concentration of inorganic phosphate in the medium is low.

**Figure 2:**
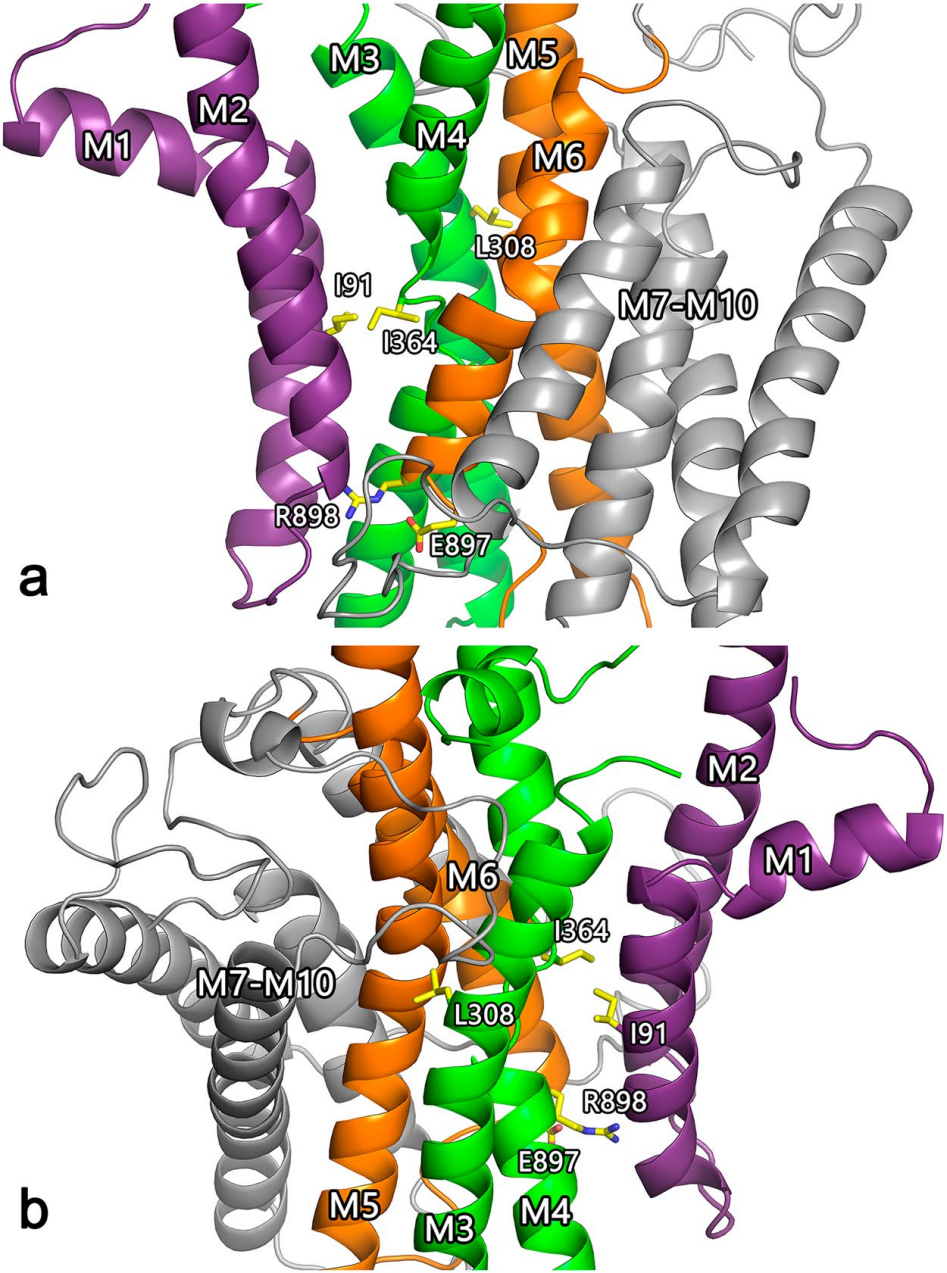
Structural model of transmembrane domains M1-M10 of bATP8A2. The M1-M2 hairpin is coloured purple, M3-M4 green, M5-M6 ochre, and M7-M10 grey. The model was previously published for both E_2_ and E_2_P states^14^ and is here shown for E_2_P in front (**a**) and back (**b**) views, respectively, along the membrane plane, with the side chains of the most important residues mentioned in the text indicated by stick representation (carbon atoms yellow, oxygen red, and nitrogen blue). The exoplasmic side is down and the cytoplasmic side up. Hence, the phospholipid is transported upwards.

Amino acid sequence comparison suggests an overall molecular structure of flippases similar to that known from X-ray crystallography of the ion-pumping prototypical P-type ATPases Ca^2+^-ATPase (SERCA) and Na^+^,K^+^-ATPase, with cytoplasmic so-called phosphorylation (P), nucleotide-binding (N) and actuator (A) domains that mediate ATP hydrolysis by catalysing formation and decomposition of a phosphorylated intermediate (Fig. 1a), and a transmembrane domain consisting of 10 helices (M1–M10), which likely mediate the transport of the specific lipid substrate^2, 14–17^. A structural model of bovine ATP8A2 (in the following abbreviated bATP8A2) was built based on the high-resolution crystal structures of SERCA in E_2_ and E_2_P states and refined by molecular dynamics simulation (Fig. 2)^14^. However, the flipping mechanism and the transport pathway for the lipid substrate, which is about 10-fold larger than the inorganic ions transported by the ion pumps, pose an enigma referred to as the giant substrate problem^14, 16, 17^. One proposed solution based on the structural modelling in combination with mutagenesis is that lipid flipping occurs by movement of the polar lipid head group in a groove on the surface of the protein, being propelled by a cluster of hydrophobic residues undergoing conformational changes, and with the lipid hydrocarbon chains following passively, still in the membrane lipid phase (“hydrophobic gate pathway”)^14^. Information on the translocation pathway has also emerged from experiments swapping PC and PS selectivity between the yeast flippases Dnf1 and Drs2^18^. However, most of the residues pinpointed in this way as determinants of the head-group selectivity and, thus, as putative interaction sites for the head group during the translocation are located closer to the cytoplasmic surface than to the exoplasmic surface at the so-called “exit gate”, which is puzzling, because the lipid is translocated from the exoplasmic leaflet toward the cytoplasmic leaflet, thus expectedly requiring a means of discriminating between different phospholipid head groups near the exoplasmic surface.

It was previously shown that bATP8A2 can be expressed in HEK293T cells and purified by immunoaffinity chromatography in sufficient amounts for detailed studies of its reaction cycle, which is activated specifically by PS and PE, but not by PC^6, 15^. With this methodology, we set out to study the functional consequences of ATP8B1 liver disease mutations by introducing them in bATP8A2. We report here results pinpointing residues of bATP8A2 that seem to interact closely with the lipid head group during the translocation process. The two juxtaposed residues E897 and R898 may be part of an exoplasmic facing “entry gate” of the translocation pathway.

## Results

### Mutants and expression

ATP8B1 mutations L127P and E981K have been reported for patients with PFIC1, whereas ATP8B1 I344F is a BRIC1 mutation^13, 19^. The equivalent mutations of bATP8A2 are I91P, E897K, and L308F. Table 1 lists these and other mutations studied in the present work. The locations in the modelled bATP8A2 E_2_P structure of the most important of the residues studied here are indicated in Fig. 2, which also indicates the position of I364, previously shown to be a critical element in the transport mechanism of human as well as bovine ATP8A2 (“hydrophobic gate residue”)^14^. All the mutant bATP8A2 proteins could be expressed together with the accessory CDC50A subunit in HEK293T cells and purified in amounts sufficient for detailed functional studies (Table 1). The lowest expression level (12% of wild type) was seen for the I91P mutant. A markedly reduced expression level relative to the wild type was seen for the mutants I91A, E897K, and E897R, as well (~30%, Table 1), whereas expression levels were higher for the other mutants including L308F, which showed wild type-like expression.

**Table 1:** Analysis of expression and ATPase activity with PS and PE.

Mutant^a^	Expression^b^		ATPase activity stimulated by PS^c^				ATPase activity stimulated by PE^c^			
	% of WT	*n*	V_max_ with PS, µmol/min/mg	*n*	K_0.5_ for PS, µM	*n*	V_max_ with PE, µmol/min/mg	*n*	K_0.5_ for PE, µM	*n*
WT	100 ± 12	14	97 ± 4	31	34 ± 1	342	50 ± 3	26	386 ± 16	264
I91P (L127P)	12 ± 2***	4	18 ± 2 (5.4x↓)***	6	96 ± 6 (2.8x↑)***	60	3 ± 0.4 (17x↓)***	2	1245 ± 522 (3.2x↑)***	20
I91A	29 ± 3**	3	88 ± 15 (1.1x↓)	7	65 ± 4 (1.9x↑)***	70	46 ± 9 (1.1x↓)	4	516 ± 48 (1.3x↑)	40
L308F (I344F)	104 ± 34	2	85 ± 1 (1.1x↓)	4	151 ± 10 (4.4x↑)***	40	28 ± 2 (1.8x↓)**	4	923 ± 65 (2.4x↑)***	40
L308A	88 ± 18	2	96 ± 5 (1.0x)	5	38 ± 2 (1.1x↑)	50	35 ± 4 (1.4x↓)	4	430 ± 18 (1.1x↑)	39
E897K (E981K)	29 ± 7*	2	19 ± 4 (5.1x↓)***	8	11 ± 1 (3.1x↓)***	78	9 ± 3 (5.6x↓)***	5	422 ± 48 (1.1x↑)	50
E897A	54 ± 18	3	41 ± 8 (2.4x↓)***	8	17 ± 1 (2.0x↓)***	79	33 ± 8 (1.5x↓)**	10	349 ± 30 (1.1x↓)	104
E897D	98 ± 5	2	92 ± 3 (1.1x↓)	4	43 ± 3 (1.3x↑)	40	50 ± 7 (1.0x)	4	495 ± 31 (1.3x↑)	39
E897Q	78 ± 10	3	60 ± 3 (1.6x↓)*	5	17 ± 1 (2.0x↓)***	50	38 ± 13 (1.3x↓)	5	255 ± 15 (1.5x↓)	50
E897F	45 ± 7	2	31 ± 4 (3.1x↓)***	4	7 ± 1 (4.9x↓)***	39	71 ± 12 (1.4x↑)*	4	476 ± 28 (1.2x↑)	39
E897R	29 ± 1*	2	10 ± 1 (9.7x↓)***	4	13 ± 1 (2.6x↓)***	40	3 ± 0.1 (17x↓)***	5	1205 ± 59 (3.1x↑)***	50
R898A	66 ± 8	5	76 ± 12 (1.3x↓)	8	53 ± 3 (1.6x↑)***	80	27 ± 3 (1.9x↓)***	9	353 ± 30 (1.1x↓)	90
R898D	89 ± 12	3	83 ± 11 (1.2x↓)	5	126 ± 4 (3.7x↑)***	60	9 ± 0.3 (5.6x↓)***	4	1069 ± 140 (2.8x↑)***	39
R898E	82 ± 13	2	59 ± 13 (1.6x↓)**	6	97 ± 5 (2.9x↑)***	70	12 ± 4 (4.2x↓)***	5	719 ± 93 (1.9x↑)***	50
R898K	79 ± 5	2	105 ± 22 (1.1x↑)	4	30 ± 1 (1.1x↓)	50	43 ± 7 (1.2x↓)	4	591 ± 49 (1.5x↑)	50
E897R_R898E	56 ± 1	2	35 ± 2 (2.8x↓)***	4	52 ± 3 (1.5x↑)***	40	11 ± 5 (4.5x↓)***	4	888 ± 37 (2.3x↑)***	40
D99A	85 ± 2	2	85 ± 14 (1.1x↓)	6	40 ± 1 (1.2x↑)	40	17 ± 4 (2.9x↓)***	4	541 ± 29 (1.4x↑)	40
R105A	62 ± 9	2	48 ± 6 (2.0x↓)**	4	45 ± 3 (1.3x↑)**	79	25 ± 2 (2.0x↓)*	3	487 ± 47 (1.3x↑)	27

**Figure 3:**
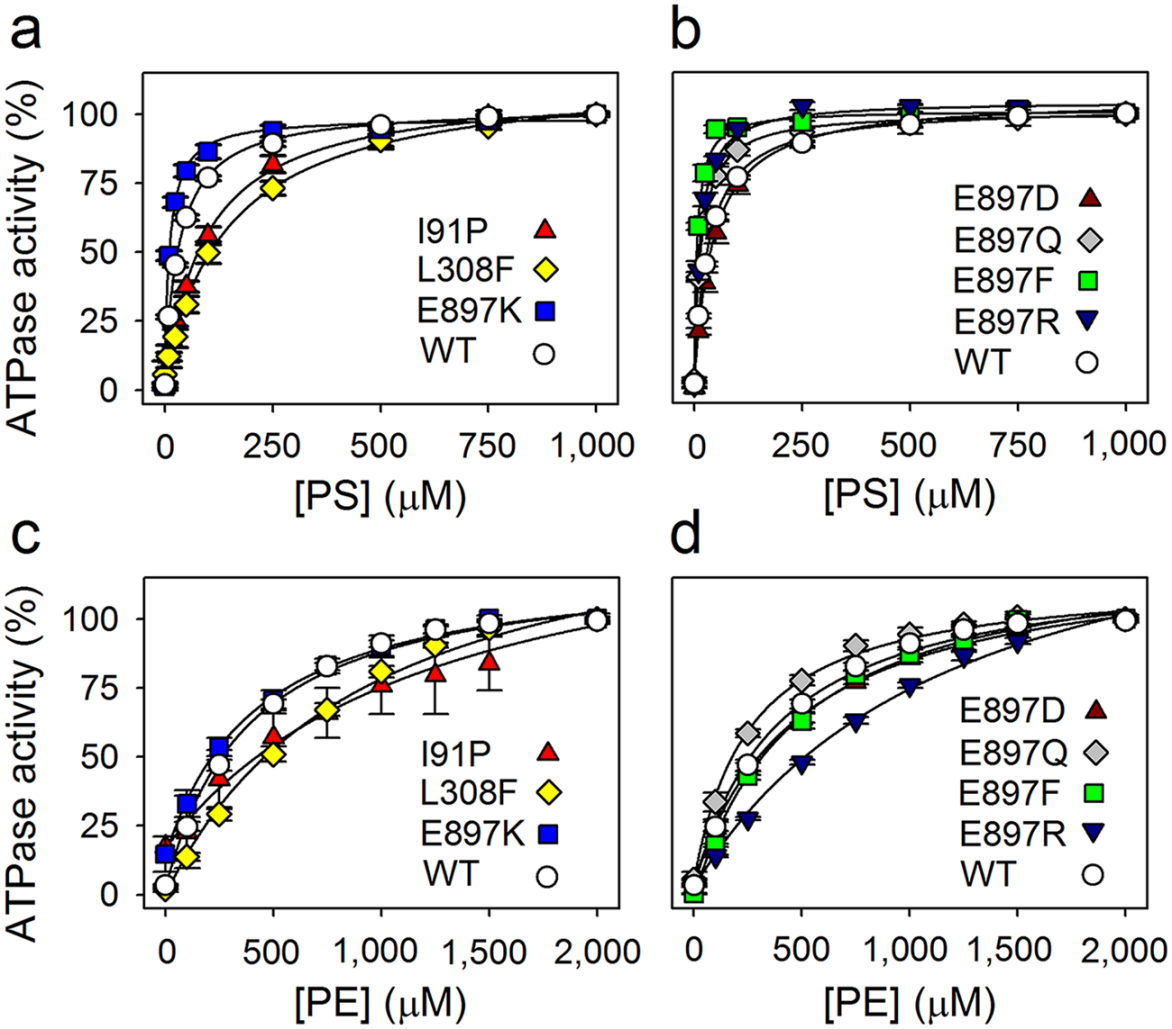
Lipid substrate dependence of ATPase activity of disease mutants and additional E897 mutants. The ATPase activity of the wild type bATP8A2 (WT) and the indicated mutants was determined at the indicated concentrations of PS (**a**, **b**) or PE (**c**, **d**). The total lipid concentration was fixed at 2.5 mg/mL (the remainder constituted by PC). The activity is shown as % of V_max_ (the absolute value of V_max_ is given in Table 1). The lines represent best fits of the Hill equation V/V_max_ = [L]^H^/(K_0.5_^H^ + [L]^H^), where the ligand L is either PS or PE. The Hill coefficient H resulting from the regression was ∼1 in all cases. The extracted K_0.5_ values and statistics are shown in Table 1.

**Figure 4:**
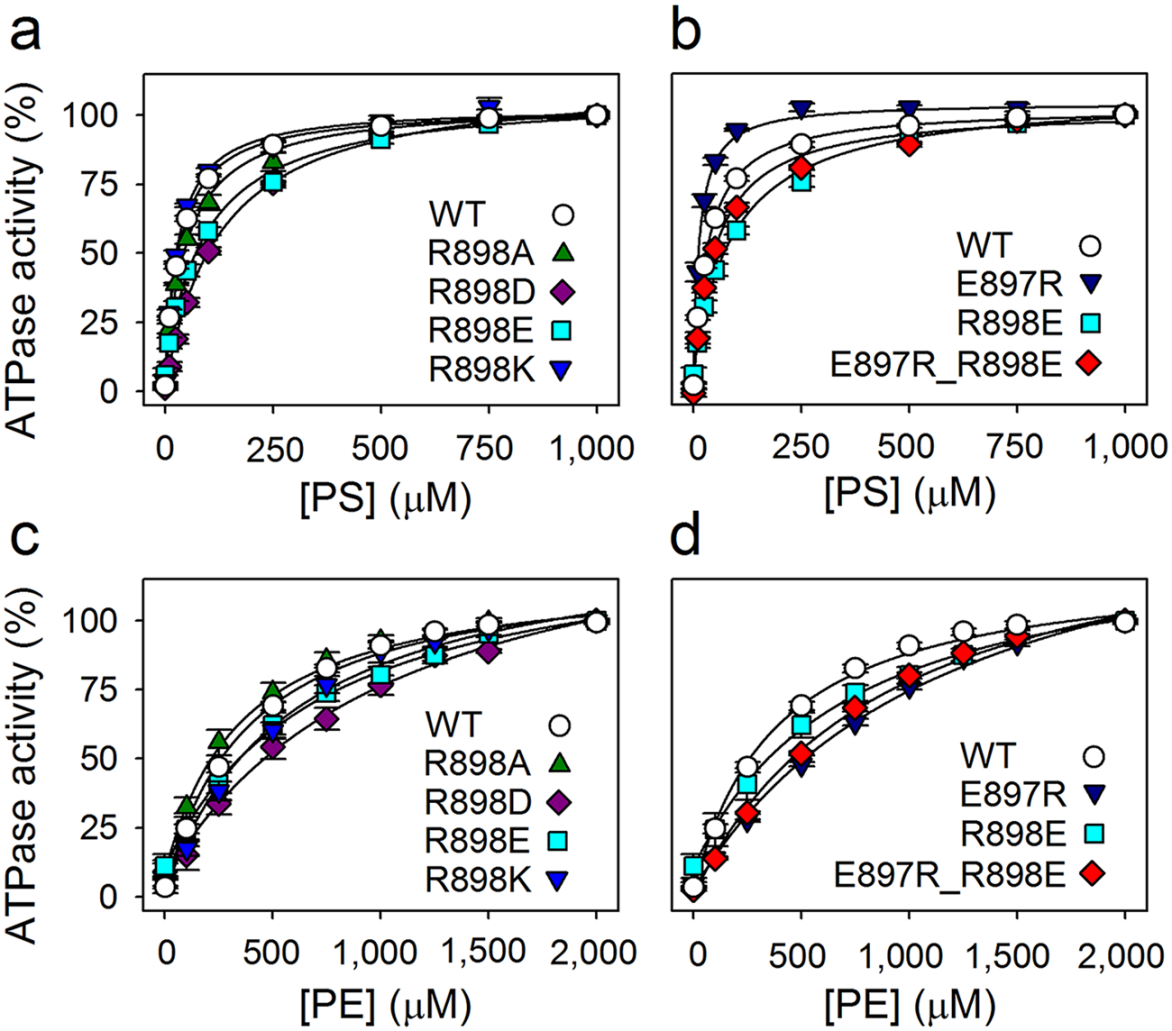
Lipid substrate dependence of ATPase activity of R898 mutants. The ATPase activity of the wild type bATP8A2 (WT) and the indicated mutants was determined at the indicated concentrations of PS (**a**, **b**) or PE (**c**, **d**). The data for R898E and E897R are repeated in (**b** and **d**) for direct comparison with E897R_R898E. Further information as in the legend to Fig. 3.

Data deviating significantly from WT according to the one-way ANOVA test (see further in Methods) are marked with *(0.05 > P > 0.01), **(0.001 < P < 0.01), and ***(P < 0.001). ^a^Wild type (WT) and mutant bATP8A2 proteins. Homologous disease mutations in human ATP8B1 are indicated in parentheses. ^b^Average of expression levels calculated relative to the wild type of the same transfection experiment, *n* indicating the number of transfection experiments. ^c^V_max_ refers to the ATPase activity at the highest lipid concentration examined (1000 µM PS or 2000 µM PE), K_0.5_ (substrate concentration giving half maximum activation) was obtained by nonlinear regression curve fitting (see Figs 3 and 4), *n* indicating the number of data points in the regressio*n*. In parentheses is shown fold change relative to wild type (arrow pointing upward/downward for increase/decrease).

### Maximal ATPase activity and apparent PS and PE lipid affinities

The expressed bATP8A2/CDC50A protein complexes were purified by detergent solubilization and immunoaffinity chromatography and reconstituted in vesicular form with PC as the only lipid present by detergent removal by dialysis. For assay of ATPase activity, the reconstituted proteoliposomes were solubilized with CHAPS detergent, and various concentrations of PS or PE (together with PC, keeping the total lipid concentration constant) were added, as well as ATP. This procedure allowed determination of the maximal rate of ATP hydrolysis per mg of pure bATP8A2 protein (V_max_) and the apparent affinities for PS and PE (as measured by the K_0.5_ values for stimulation of the ATP hydrolysis by the lipid, Figs 3 and 4, Table 1). Under optimal conditions at saturating PS concentration and 37 °C the V_max_ of the wild type bATP8A2 was 97 μmol/min/mg bATP8A2 protein (average of measurements on 14 preparations over more than a year), corresponding to a turnover rate (k_cat_) of 209 s^–1^. This value is of the same order of magnitude as the maximal turnover rates determined for the ion-pumping P-type ATPases Ca^2+^-ATPase (SERCA) and Na^+^,K^+^-ATPase, but is 1–2 orders of magnitude higher than the turnover rates that have been measured for the yeast homolog Drs2, which seems, at least in part, due to the longer, auto-inhibitory C-terminus of Drs2^2, 20^. Like the wild type bATP8A2 protein, all the mutants required the presence of PS or PE for activation, PS being the preferred substrate with higher affinity and giving higher V_max_ than PE. Only diminutive ATPase activity was seen with PC alone (at approx. 3000 μM PC the activity of WT and all mutants was less than 6% of the maximal PS-stimulated activity, data points at 0 μM PS in Figs 3 and 4).

The I91P mutation led to a dramatic reduction of the activity, the V_max_ being ~5-fold reduced with PS as the activating lipid and ~17-fold with PE, relative to the wild type, whereas the V_max_ was wild type-like for I91A with either lipid. The apparent affinity of I91P was ~3-fold reduced (3-fold increased K_0.5_) for both PS and PE, whereas the reduction of the apparent affinity was less pronounced for I91A (Fig. 3a,c and Table 1).

Replacement of L308 with the even larger hydrophobic phenylalanine side chain led to a wild type-like V_max_ with PS as the activating lipid, but the apparent affinity was notably reduced for both lipid substrates, ~4-fold for PS and ~2-fold for PE, as was the maximal rate with PE (~2-fold, Fig. 3a,c and Table 1). The L308A mutant showed more wild type-like functional properties compared with L308F (Table 1).

The E897K mutation resulted in marked ~5- and ~6-fold reductions of V_max_ with PS and PE as activating substrates, respectively. Moreover, mutation E897K caused a ~3-fold increase of the apparent affinity for PS relative to wild type (3-fold reduced K_0.5_), but without significant effect on the apparent affinity for PE (Fig. 3a,c and Table 1). For further elucidation of the functional role of the negatively charged E897 side chain, additional mutations to E897 were introduced: E897A, E897D, E897Q, E897F, and E897R. The latter mutation resulted in dramatic 10- and 17-fold reductions of V_max_ with PS and PE as activating substrates, respectively, whereas the effects on V_max_ were much less pronounced for the other E897 mutants. The E897D mutant, retaining a carboxylate group in the side chain, was rather wild type-like for all the parameters determined. As with E897K, significant increases of the apparent affinity for PS (reduced K_0.5_), relative to wild type, were observed for the mutants E897A (~2-fold), E897Q (~2-fold), E897F (~5-fold), and E897R (~3-fold), whereas the apparent PE affinity was wild type-like or less affected than the apparent PS affinity, or even reduced (E897R), see Fig. 3b,d and Table 1.

Mutants with alterations to the neighbouring arginine, R898, were also analysed (Fig. 4 and Table 1), showing insignificant or rather moderate (R898E) reductions of V_max_ with PS as the activating lipid and larger reductions of V_max_ with PE, particularly the R898D and R898E mutants where the positively charged arginine side chain is replaced by a negatively charged carboxylate side chain. These mutations also markedly reduced the apparent affinity for both lipid substrates (K_0.5_ increased 3- to 4-fold for PS and 2- to 3-fold for PE). The alanine substitution reduced the apparent affinity only for PS (~2-fold).

**Figure 5:**
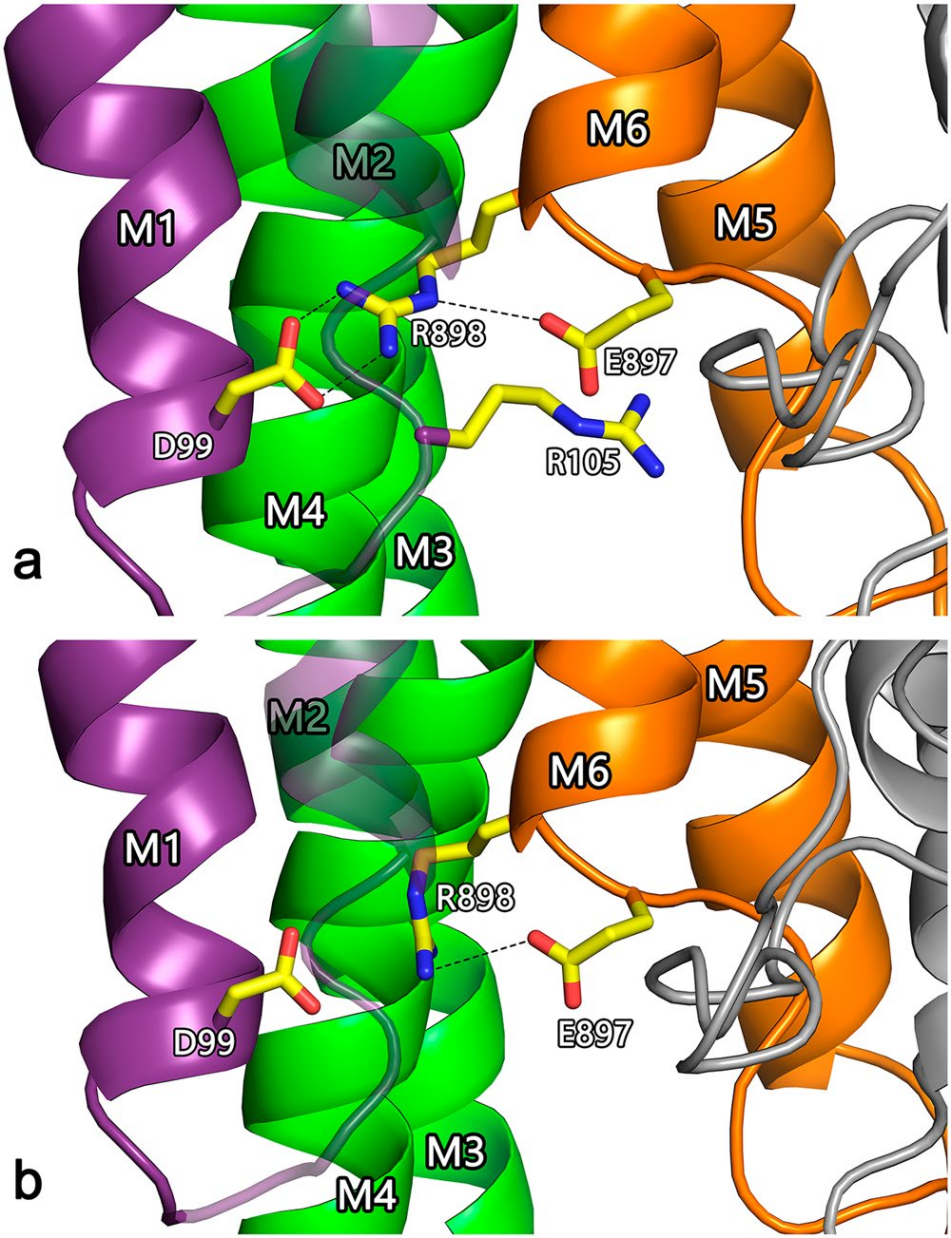
Close up view of the E897-R898 region with alternative R898 side chain rotamers. Side chains of E897 and R898, as well as the additional residues examined in this study, D99 and R105 (only shown in **a**), are indicated by stick representation. Dotted lines indicate distances shorter than 3.6 Å, i.e. compatible with a salt bridge. The shortest distance between R105 guanidine-N and E897 side chain-O is 5.0 Å. (**a**) Originally modelled rotamer of R898 side chain (same as in Fig. 2). (**b**) Alternative R898 rotamer, where no salt bridge is formed with D99. The overall structural model and colour codes for membrane helices and side chains are the same as in Fig. 2.

A swap mutant, E897R_R898E, was constructed to examine the possibility that E897 and R898 interact through a salt bridge (see Fig. 5). An essential finding with this double mutant was that the R898E mutation compensates the drastic 10- and 17-fold reductions of V_max_ with PS and PE, respectively, observed for the single mutation E897R, thus resulting in only ~3- and ~5-fold reductions of V_max_, respectively, in the swap mutant, even though the point mutation R898E by itself reduces V_max_. Furthermore, the apparent PS affinity of the E897R_R898E mutant deviated less (1.5-fold reduction) from that of the wild type than the PS affinities of the singly substituted mutants E897R (2.6-fold increase) and R898E (2.9-fold reduction). The apparent PE affinity of the swap mutant was 2.3-fold reduced, again being intermediate between E897R and R898E (3.1- and 1.9-fold reduced, respectively, Table 1).

The mutations D99A and R105A were also introduced in bATP8A2 and studied, because the structural model suggests potentially important interactions of D99 and R105 with R898 (see Discussion and Fig. 5a). For the D99A mutant, the V_max_ with PS as activating lipid and the apparent PS and PE affinities were wild type-like, whereas V_max_ with PE was ~3-fold reduced, relative to the wild type. For the R105A mutant, the V_max_ was reduced ~2-fold, both with PS and with PE as activating lipid. The effects of mutation R105A on the apparent lipid affinities were slight, 1.3-fold reduction for PS, and for PE the same was seen, although in the latter case the difference from wild type was not statistically significant (Table 1).

## Discussion

Of the three studied bATP8A2 mutations equivalent to ATP8B1 disease mutations, I91P and E897K most strongly affected the expression level and the V_max_, whereas the L308F mutation reduced the apparent affinities for the PS and PE lipid substrates markedly, but did not reduce the expression, and exerted no (for PS) or less (for PE) effect on the V_max_ than I91P and E897K. Because a selective reduction of affinity may allow partial retention of the lipid flipping activity under physiological conditions, the observed differences between the mutants seem consistent with the clinical courses of the liver disease in the patients harbouring the ATP8B1 mutations: ATP8B1 L127P and E981K, equivalent to bATP8A2 I91P and E897K, give rise to PFIC1, which has a very severe clinical course, and ATP8B1 I344F, equivalent to bATP8A2 L308F, causes BRIC1, which has a milder clinical course.

Just like wild type bATP8A2, the bATP8A2 mutants studied required PS or PE to activate ATP hydrolysis, and no appreciable activation occurred with PC alone up to approx. 3000 μM, i.e. higher than the concentrations of PS and PE required for maximal activity. Hence, there is no indication that the mutations converted bATP8A2 to a PC flippase. Nevertheless, some of the observed mutational effects on the PS and PE stimulated ATPase activity of bATP8A2 are largely similar to the previously described effects of the equivalent mutations in ATP8B1 and yeast Dnf2 on their PC flippase activity measured as uptake of fluorescent NBD-labelled PC in whole cells^8, 21^ (Table 2). Hence, the previous study demonstrating uptake of NBD-PC in HeLa cells expressing wild-type ATP8B1 also showed that either of the ATP8B1 mutations L127P and I344F abolishes NBD-PC uptake, although no distinction between the effects on V_max_ and apparent affinity was possible in this study, because only one probe concentration was tested^8^. In the study of NBD-PC uptake by yeast Dnf2 mutants^21^, the apparent lipid affinity was determined and was found reduced in the N601F mutant (equivalent to bATP8A2 L308F and ATP8B1 I344F) and enhanced in E1261K (equivalent to bATP8A2 E897K and ATP8B1 E981K), the latter mutant also showing reduced V_max_. These similarities between the effects of equivalent mutations are remarkable, considering that different flippase proteins, lipid substrates, and expression systems were studied. However, a wild type-like expression and function was reported for yeast Dnf2 L264P^21^, which differs from the robust effects of the corresponding bATP8A2 I91P and ATP8B1 L127P mutations (Table 2).

**Table 2:** Summary of present results on disease mutants and comparison with previous studies.

	Present study (bATP8A2, PS)^a^			Ref. 8 (ATP8B1, PC)^b^			Ref. 21 (Dnf2, PC)^c^		
Mutant	I91P	L308F	E897K	L127P	I344F	E981K	L264P	N601F	E1261K
Expression	↓	WT	↓	↓	WT	n.d.	WT	WT	WT
V_max_	↓	WT	↓	Lipid uptake ↓	Lipid uptake ↓		WT	WT	↓
Apparent affinity for lipid	↓	↓	↑				WT	↓	↑

The mutational effects indicated refer to measurements of ATPase activity stimulated by PS^a^ or uptake of fluorescently labelled PC (substrate of ATP8B1 and Dnf2) in mammalian^b^ or yeast^c^ cells. A ≥1.5-fold change is considered different from wild type and is indicated by arrow pointing upward/downward for increase/decrease (WT = wild type-like). L127P, I344F, and E981K are disease mutations in ATP8B1 identified in patients. The equivalent mutations in the bATP8A2 studied here are I91P, L308F, and E897K, and in yeast Dnf2 L264P, N601F, and E1261K, respectively. Only a single PC concentration was tested with ATP8B1, thus precluding distinction between effects on V_max_ and K_0.5_^b^, and E981K was not studied (n.d., not determined)^8^. In the latter study, the lipid uptake was corrected for variation of ATP8B1 protein expression at the surface of the cells, thus being comparable to the V_max_ value of the present study, which is the rate of ATP hydrolysis per mg of bATP8A2 protein.

The observed dependence of the ATPase activity of bATP8A2 on the specific lipid substrates PS and PE reflects the activation of the rate-limiting dephosphorylation step (E_2_P to E_2_) by the binding of these lipids to E_2_P (see reaction cycle in Fig. 1a)^15^. To appreciate the meaning of the apparent affinities (K_0.5_ values) extracted from the data in Figs 3 and 4, it is helpful to realize that the K_0.5_ essentially is the Michaelis constant K_m_ of the reaction shown in Fig. 1b, hence

1 $${{\rm{K}}}_{{\rm{0.5}}}={{\rm{K}}}_{{\rm{m}}}={({\rm{k}}}_{{\rm{cat}}}+{{\rm{k}}}_{\mbox{--}1}{)/{\rm{k}}}_{1}$$

Because the dephosphorylation is rate limiting for the overall ATPase reaction, the k_cat_ corresponds to the maximal turnover rate (rate of ATP hydrolysis per enzyme molecule) and is directly proportional to the V_max_ (here denoting the rate of ATP hydrolysis per mg of enzyme). Hence the V_max_ is a co-determinant of the observed K_0.5_ for the phospholipid together with the dissociation constant, k_–1_/k_1_, characterizing the “true” or intrinsic affinity of E_2_P for the phospholipid. Based on equation (1) it can be concluded that in those mutants where the V_max_ (i.e. k_cat_) is normal or reduced and the K_0.5_ for the lipid substrate increased (i.e. corresponding to reduced apparent affinity), relative to wild type, the mutation must have increased k_–1_ and/or reduced k_1_, thus reducing the intrinsic affinity for the phospholipid. Such is the case for L308F, both with PS and PE as activating lipid, and it is therefore likely that this mutation disturbs a binding site used by either lipid during the translocation. In our bATP8A2 structural model (Fig. 2) the L308 side chain projects into the interface between the M3 and M5 helices close to the region proposed as “exit gate”^18, 22^, and the bulky phenylalanine substituent may therefore have interfered directly with the binding of the lipid substrate here. In the hydrophobic gate pathway hypothesis^14^ the disturbance would be more indirect, perhaps being caused by perturbation of the interaction between M3 and M5 or the interaction with the accessory subunit known to play a role in the binding of the lipid substrate^21, 23^. In the study of the equivalent yeast Dnf2 mutant N601F a weakened interaction with the accessory subunit was in fact inferred by using a mating-based split ubiquitin assay^21^.

**Figure 6:**
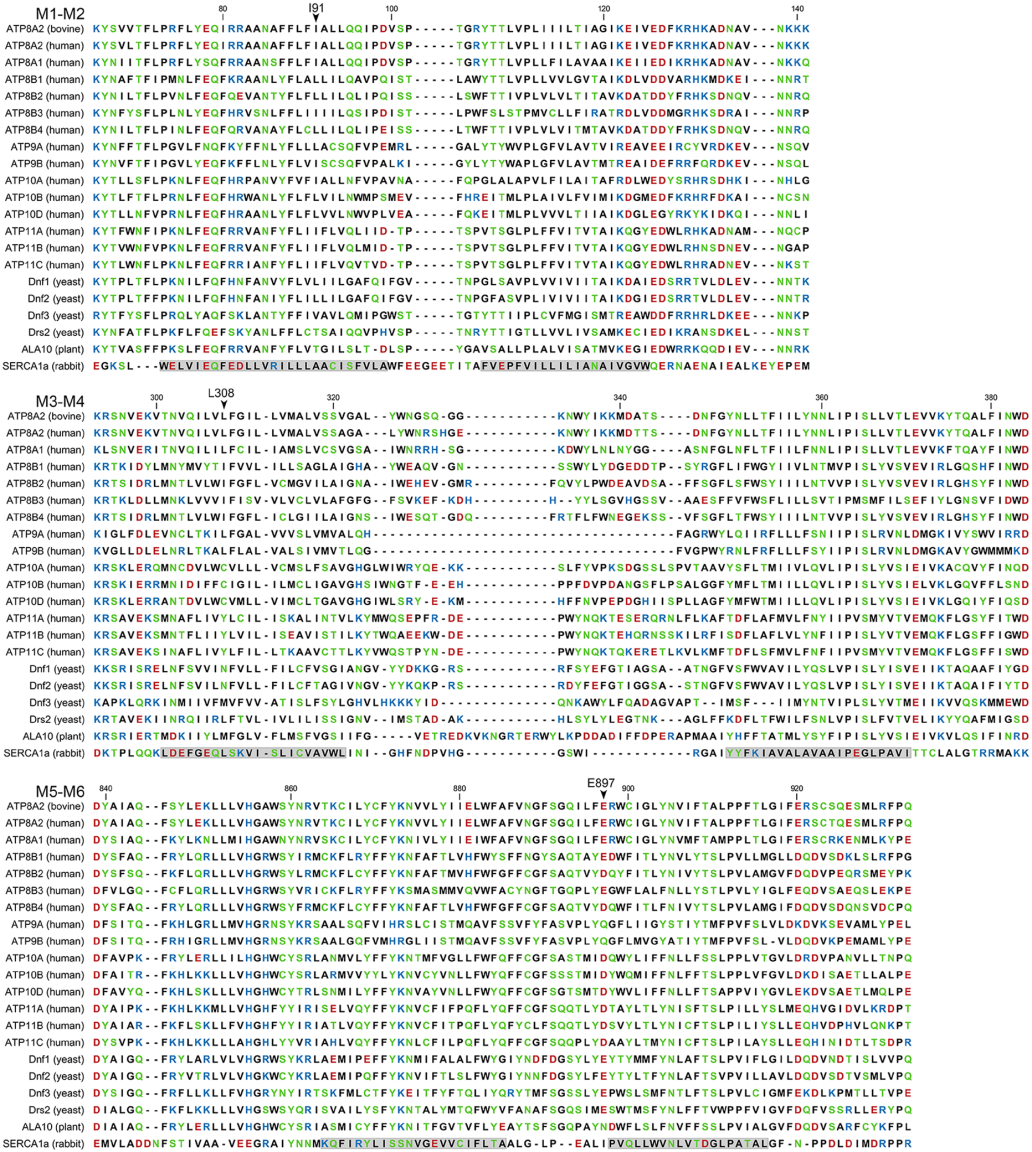
Alignment of the regions corresponding to the first six transmembrane segments of P4-ATPases and SERCA. Protein sequences of the bovine ATP8A2 studied here, the 14 human flippases, 4 yeast flippases, and one plant flippase (UniProt nos. from above: C7EXK4, Q9NTI2, Q9Y2Q0, O43520, P98198, O60423, Q8TF62, O75110, O43861, O60312, O94823, Q9P241, P98196, Q9Y2G3, Q8NB49, P32660, Q12675, Q12674, P39524, and Q9LI83) were aligned with rabbit SERCA1 Ca^2+^-ATPase (UniProt no. P04191) as previously described^14^. Letter colours denote positively (blue) or negatively (red) charged, polar (green), or hydrophobic (black) residues. The residues replaced in the three disease mutants studied here are indicated with arrowheads. Numbering refers to the bovine ATP8A2. Grey shading denotes the transmembrane helices identified in SERCA crystal structures. The human P4-ATPases are subdivided into classes of genetically most related proteins: Class 1a (ATP8A1, ATP8A2), Class 1b (ATP8B1, ATP8B2, ATP8B3, ATP8B4), Class 2 (ATP9A, ATP9B), Class 5 (ATP10A, ATP10B, ATP10D), and Class 6 (ATP11A, ATP11B, ATP11C)^2^.

Following the same analysis as outlined above, the reduced apparent affinity of the I91P mutant for PS and PE in conjunction with its reduced V_max_ values, relative to wild type, suggests that the intrinsic affinity for either phospholipid substrate is reduced in I91P. Interestingly, the side chain of I91 is within 3 Å of I364 of M4 in the E_2_P model (Fig. 2). I364 is the central residue in the proposed hydrophobic gate pathway for lipid translocation, the position of I364 in the amino acid sequence being equivalent to that of the M4 glutamate that binds and translocates the cations in Ca^2+^- and Na^+^,K^+^-ATPases^14^. I91 is conserved as isoleucine or leucine in the majority of the P4-ATPases (see Fig. 6) and might well participate in hydrophobic interaction in the hydrophobic gate, which would be disturbed by the I91P mutation, thus explaining the effect on affinity as well as V_max_.

The glutamate E897 is located in the exoplasmic loop between M5 and M6 at the border of M6 (Figs 2 and 5). The E897K mutation reduced both the V_max_ and the K_0.5_ for PS, raising the possibility that the reduced K_0.5_ is a consequence of the reduced k_cat_ rather than of an increase of the intrinsic affinity for PS. However, the relative change of V_max_ imposed by this mutation is very similar for PS and PE (5- to 6-fold), and yet the K_0.5_ for PE was essentially unchanged (Table 1), which leads us to speculate that most likely the E897K mutation selectively increased the “true” affinity for PS relative to PE. A similar explanation seems to hold for the other E897 mutants except E897D, which was wild type-like, suggesting that the negatively charged carboxylate group is more important than the length of the side chain. The difference between PS and PE is particularly obvious for the E897R mutant, which showed reduced K_0.5_ with PS as activating lipid and increased K_0.5_ with PE, despite a large reduction of V_max_ in both cases. Taken together, the data support the hypothesis that the lipid substrate passes relatively close to the E897 side chain during the transport process in the wild type bATP8A2, such that the net negatively charged PS head group senses the negative charge of E897 (or aspartate as substituent), thus being repelled in contrast to the neutral PE head group. Mutations to E897 that remove the negative charge or introduce a positive charge interacting favorably with the negatively charged PS head group will therefore lead to increased affinity. The reduced apparent affinity for PE seen for the E897R substitution could possibly arise from partial steric hindrance by the bulky arginine side chain of the access of the lipid head group to a binding site/exoplasmic entry of the transport pathway. In the case of the PS head group, such steric hindrance might be compensated by the electrostatic attraction to the positively charged arginine side chain.

It is of note that the K_0.5_ for PS is reduced more in the E897F mutant (∼5-fold) than in any of the other E897 mutants, still with no reduction of the K_0.5_ for PE. Hence, the insertion of an aromatic side chain at this position not only prevents electrostatic repulsion of PS by E897, but also in some way increases the likelihood that PS will bind.

Further, according to the considerations above (i.e. equation (1)), the reduced apparent affinity for PS and PE combined with wild type-like or reduced V_max_ seen upon replacement of R898 with aspartate or glutamate indicates that these charge-reversing substitutions reduce the intrinsic affinity for either lipid. However, the effect was strongest for PS, and the mere removal of the positive charge by the R898A mutation reduced only the PS affinity. In addition, little effect was seen for the R898K mutation that retains the positive charge of the side chain. Hence, the most reasonable interpretation is that in the wild type the positive charge of the arginine side chain interacts favourably with the negatively charged head group of PS during the transport process, and that a negatively charged side chain at this position repels not only PS, but also to some extent PE, probably due to closeness to its local negative charge at the phosphate group.

Because interaction of R898 with E897 through a salt bridge would be feasible according to the molecular modelling (Fig. 5a,b), we examined the swap mutant E897R_R898E. Separately, both of the mutations E897R and R898E reduced V_max_ with either PS or PE as activating lipid, thus it might have been expected that the double mutant would be further inhibited. However, the maximal rate determined for E897R_R898E, either with PS or PE, is intermediate between the individual rates of the point mutants, in fact equal to or close to the calculated average of these rates (Table 1, example for V_max_ with PS: the average of 10 (for E897R) and 59 (for R898E) is 34.5, compare with 35 (for E897R_R898E)), which means that R898E results in a gain-of-function when present together with E897R, contrary to its effect alone. Hence, E897 and R898 seem to interact, and this interaction is partly preserved when the residues swap positions. Collectively, the data obtained with the individual E897 and R898 mutants and the swap mutant suggest that the lipid substrate comes close to the location of these residues during the transport process, sensing the charges of both, and that these residues also interact with each other through a salt bridge. Another possible player in this region is the arginine R105 located in the exoplasmic loop between M1 and M2 (Fig. 5a). Indeed the slight reduction of the apparent affinity for the lipid substrate in combination with reduced V_max_ seen for mutation R105A shows that the lipid substrate senses R105, although less strongly than R898.

In the structural model (Fig. 5a) the side chain of R898 is sufficiently close to the aspartate D99 of M1 to allow formation of a salt bridge between them. However, the wild type-like V_max_ in the presence of PS and wild type-like PS affinity of the D99A mutant (Table 1) indicate that such interaction is unimportant for PS binding. The model does not include the lipid substrate and, hence, does not show the structural adaptations to its binding. The actual rotamer of the R898 side chain may thus be different from that of the original model^14^, at least in the PS-bound state. As shown in Fig. 5b, an option is a rotamer forming a bond only with E897 and not with D99.

Consistent with the results of the functional analysis, the location of E897 and R898 in the exoplasmic M5-M6 loop at the border of M6 would allow these residues to be among the first residues encountered by lipids destined for translocation, thus being part of an entry gate that contributes to selection of the lipid. The salt bridge between E897 and R898 could be important in preventing the negative charge of the E897 side chain from repelling the PS head group, thereby ensuring high affinity for PS. While glutamate or aspartate is present at the position corresponding to bATP8A2 E897 in most P4-ATPases, the juxtaposed positively charged arginine is unique to ATP8A1 and ATP8A2 (Fig. 6), which both have high specificity for PS. The PC flippase ATP8B1 has a pair of anionic carboxylic acid residues, E981 and D982, at the corresponding positions. According to the present results with R898D, the aspartate will markedly reduce the affinity for PS (Table 1). Therefore the aspartate may contribute to ensure PC selectivity of ATP8B1. On the other hand, not all PS flippases have a positively charged residue at the position corresponding to R898 in bATP8A2. A neutral residue is found in ATP11A and ATP11C (threonine and alanine, respectively), which both are well documented PS flippases^8^. The yeast PS flippase Drs2 likewise has a neutral residue (serine) at this position, and the yeast PC flippases Dnf1 and Dnf2 also have a neutral residue (tyrosine) here (Fig. 6). Thus, there is no clear correlation between the substrate specificity and the charge of the side chain at this position all through the P4-ATPase family members. Variation here might in fact be a physiologically important means of endowing the various PS flippases with differential PS affinity. To this can be further added the variation of the residue at the position corresponding bATP8A2 L308. The mammalian P4-ATPase classes each have their characteristic amino acid at this position: leucine for Class 1a (ATP8A1,A2), isoleucine for Class 1b (ATP8B1,B2,B3,B4), leucine for Class 2 (ATP9A,B), cysteine for Class 5 (ATP10A,B,D), and tyrosine for Class 6 (ATP11A,B,C) (Fig. 6, see class nomenclature in figure legend). Based on the present data for the mutants L308F and R898D (Table 1), the PS affinity of L308F is even lower than that of R898D. Due to the similar chemical properties of the tyrosine and phenylalanine side chains, the tyrosine present at this position in Class 6 flippases would be expected to further contribute to lower their PS affinity relative to Class 1a. A future challenge is therefore to experimentally determine the affinity of Class 6 flippases for the lipid substrate.

## Methods

### Flippase enzyme

The enzyme studied here is bovine ATP8A2 (bATP8A2, EC 3.6.3.1, UniProt C7EXK4). The applied procedures for mutagenesis, expression, purification, and reconstitution in lipid vesicles of wild type and mutant bATP8A2 with the accessory CDC50 subunit have previously been described in detail^6, 14, 15^. Briefly, selected mutations were introduced into bATP8A2 cDNA contained in a pcDNA3 plasmid vector by PCR. All constructs were verified by sequencing of the entire coding sequence. HEK293T cells were co-transfected with bATP8A2 and CDC50A cDNA. Cells were lysed and the flippase bATP8A2/CDC50A protein complex was immunoaffinity-purified using the Rho 1D4 antibody, which specifically recognizes a C-terminal nine amino acid 1D4 tag on bATP8A2^24^. Reconstitution of mutated or wild type bATP8A2/CDC50A in PC-containing vesicles by removal of detergent by dialysis made the protein suitable for storage.

### ATPase activity measurements

To determine the lipid substrate dependence of ATPase activity, the proteoliposomes were re-dissolved in assay buffer (9 mM CHAPS, 50 mM HEPES-NaOH (pH 7.5), 150 mM NaCl, 11.75 mM MgCl_2_ and 1 mM DTT). PS (0–1000 µM) or PE (0–2000 µM) was added together with PC (maintaining a fixed total lipid concentration of 2.5 mg/mL). The lipids, PS (1,2-dioleoyl-sn-glycero-3-phospho-serine, 18:1 PS, cat. no. 840035), PE (1,2-dioleoyl-sn-glycero-3-phospho-ethanolamine, 18:1 (Δ9-Cis) PE, cat. no. 850725), and PC (L-α-phosphatidylcholine, egg lecithin from chicken, >99% pure, cat. no. 840051), were from Avanti Polar Lipids (Alabaster, AL). The ATPase reaction (running for 15 min at 37 °C, linear time dependence in this range) was started by addition of ATP (7.5 mM) and terminated with SDS (12%). The amount of liberated phosphate was quantified by colorimetric analysis^6^.

### Data analysis and statistics

The K_0.5_ values for activation by the lipid substrates were extracted by curve fitting using the SigmaPlot program (SPSS, Inc.) for nonlinear regression (see legend to Fig. 3 for equation used). P-values for comparison of wild type and mutant regressions, expression and V_max_ values were calculated with SigmaPlot using the one-way ANOVA for multiple comparisons versus control group with Holm-Sidak test. Error bars in figures and ± in tables indicate standard error (s.e.m.). Structural figures were prepared using PyMOL software.

### Data availability statement

All source data for figures and tables in the present article are available from the corresponding author on reasonable request.
